# ConvFormer-KDE: A Long-Term Point–Interval Prediction Framework for PM_2.5_ Based on Multi-Source Spatial and Temporal Data

**DOI:** 10.3390/toxics12080554

**Published:** 2024-07-30

**Authors:** Shaofu Lin, Yuying Zhang, Xingjia Fei, Xiliang Liu, Qiang Mei

**Affiliations:** 1 Faculty of Information Technology, Beijing University of Technology, Beijing 100124, China; linshaofu@bjut.edu.cn (S.L.); zhangyuying@emails.bjut.edu.cn (Y.Z.); feixingjia@emails.bjut.edu.cn (X.F.); 2 Navigation College, Jimei University, Xiamen 361021, China; meiqiang@jmu.edu.cn

**Keywords:** fine particulate matter, long-term point prediction, interval prediction, convolutional neural network, transformer, kernel density estimation

## Abstract

Accurate long-term PM_2.5_ prediction is crucial for environmental management and public health. However, previous studies have mainly focused on short-term air quality point predictions, neglecting the importance of accurately predicting the long-term trends of PM_2.5_ and studying the uncertainty of PM_2.5_ concentration changes. The traditional approaches have limitations in capturing nonlinear relationships and complex dynamic patterns in time series, and they often overlook the credibility of prediction results in practical applications. Therefore, there is still much room for improvement in long-term prediction of PM_2.5_. This study proposes a novel long-term point and interval prediction framework for urban air quality based on multi-source spatial and temporal data, which further quantifies the uncertainty and volatility of the prediction based on the accurate PM_2.5_ point prediction. In this model, firstly, multi-source datasets from multiple monitoring stations are preprocessed. Subsequently, spatial clustering of stations based on POI data is performed to filter out strongly correlated stations, and feature selection is performed to eliminate redundant features. In this paper, the ConvFormer-KDE model is presented, whereby local patterns and short-term dependencies among multivariate variables are mined through a convolutional neural network (CNN), long-term dependencies among time-series data are extracted using the Transformer model, and a direct multi-output strategy is employed to realize the long-term point prediction of PM_2.5_ concentration. KDE is utilized to derive prediction intervals for PM_2.5_ concentration at confidence levels of 85%, 90%, and 95%, respectively, reflecting the uncertainty inherent in long-term trends of PM_2.5_. The performance of ConvFormer-KDE was compared with a list of advanced models. Experimental results showed that ConvFormer-KDE outperformed baseline models in long-term point- and interval-prediction tasks for PM_2.5_. The ConvFormer-KDE can provide a valuable early warning basis for future PM_2.5_ changes from the aspects of point and interval prediction.

## 1. Introduction

With the rapid development of industrialization and urbanization, air quality issues have become the focus of social concern, especially in rapidly developing urban areas [1]. PM_2.5_, a major factor affecting air quality, presents a serious threat to public health and environmental conservation [2]. Thus, accurate prediction of PM_2.5_ concentration is crucial for both government agencies and the public. The variations in PM_2.5_ concentrations are significantly influenced by socio-economic factors, human activities, and the spatial distribution of urban structures. Currently, most cities nationwide (such as Beijing, Guangzhou, and Haikou) have established air monitoring stations for monitoring hourly data on various air pollutants and meteorological factors [3]. However, although these monitoring stations can provide real-time air pollution data, they cannot predict pollutant concentrations in advance. Consequently, accurate advance prediction of PM_2.5_ concentration has become essential for managing environmental health and preventing severe pollution events.

Previous studies on the prediction of PM_2.5_ concentration have mainly emphasized short-term point prediction. This approach focuses solely on specific momentary values of air pollutant concentration, overlooking the long-term trends and predictive uncertainty of PM_2.5_ concentration [4,5]. Such short-term point-prediction methods pose challenges in offering comprehensive information for decision making and constrain the deep comprehension of future air quality conditions [6]. Therefore, it is particularly important to develop an air quality prediction framework that can simultaneously consider long-term point and interval prediction. However, this is still a challenging topic, and its core issues can be summarized as follows:

(1) How to fully exploit the interactions and impacts among air pollutants, meteorological factors, and spatial and temporal factors [7,8]. Meteorological factors have an important influence on the formation, transport, and dispersion of air pollutants. In addition, there is a degree of correlation between different monitoring stations. Therefore, it is crucial to fully consider the correlation between multiple monitoring stations and exploit the effects between multiple air pollutants and meteorological factors in the air quality prediction modelling process.
(2) How to improve the accuracy and reliability of long-term predictions. Accurate long-term predictions can provide us with sufficient time to take measures against air pollution. However, there are complex nonlinear relationships among the factors affecting air pollutants, and the current prediction models applied to air pollution are mainly designed for short-term prediction tasks, which makes it challenging to capture the long-term dependences among air pollution time series effectively [9]. Therefore, fully exploiting the spatial and temporal effects between air pollutant concentration and meteorological factors is the key to achieving accurate PM_2.5_ prediction.
(3) How to effectively use interval prediction to quantify uncertainty in PM_2.5_ concentration changes. Most previous studies on PM_2.5_ concentration prediction have focused on point prediction, but point prediction often has difficulty covering more fluctuating information (e.g., uncertainty, variability, and trends) [10]. The key to achieving interval prediction is modelling the point-prediction error distribution. Therefore, choosing an appropriate method to fit the point-prediction error distribution is the key to achieving interval prediction.

As we all know, the formation and variation of PM_2.5_ concentration are influenced by multiple factors, including meteorological conditions, environmental parameters, and human activities. For instance, specific meteorological conditions such as temperature and wind speed can significantly impact not only the transport and dispersion of pollutants but also determine the stability and reactivity of pollutants in the air [11]. Additionally, alterations in human activities and the distribution of points of interest (POIs) can have direct or indirect impacts on air quality [12]. Pollutant emissions from these activities can lead to correlated and synergistic PM_2.5_ concentration at different monitoring stations. However, many studies currently consider only the relationship between neighboring stations in the actual geographic area, ignoring geospatial similarity [13,14]. For example, two stations may be geographically distant, but they may exhibit similar patterns [15]. Therefore, it is imperative to fully consider the geographic similarity of all stations to enhance the accuracy of air quality prediction.

In recent years, machine learning and deep learning techniques have shown significant performance in short-term prediction of PM_2.5_ [8,16,17]. However, long-term prediction tasks present a higher challenge to existing models [18]. The core of long-term prediction modelling lies in choosing a multi-step prediction strategy [19]. The strategies commonly used in the current research can be categorized into recursive strategies [20] and direct multi-output strategies [21]. Recursive prediction strategies have the advantage of incorporating the extraction of the time dependence within the predicted sequence into the modelling. However, introducing the predicted values leads to a severe error accumulation problem [22]. Conversely, the direct multi-output prediction strategy simultaneously generates predictions at multiple time points during the training process, effectively improving the prediction efficiency and mitigating the error accumulation problem [23]. However, this strategy typically relies on complex network architectures to capture long-term temporal dependencies between time series. Moreover, existing deep learning models for short-term prediction struggle to capture long-term dependencies in time series. Recently, the Transformer model has performed well in long-term time-series prediction owing to its advantages in capturing long-term dependencies, thus providing a new direction for long-term prediction of PM_2.5_ [24]. It is worth noting that although the Transformer has significant advantages in establishing remote dependencies between data, it still has limitations in dealing with complex dependencies among multiple variables [25]. In addition, this study notes that CNNs have powerful grid-data processing capabilities to capture localized patterns and features in time series effectively. Therefore, combining a CNN with the Transformer model to construct a hybrid prediction framework that can effectively integrate multivariate and deeply mine long-term dependencies is crucial for improving the accuracy and reliability of PM_2.5_ prediction.

Although point prediction of PM_2.5_ concentration plays an important role in air pollution control, errors are inevitable in this type of prediction due to the volatility and non-stationarity of changes in PM_2.5_ concentration [6]. In order to fully consider more uncertain information, interval prediction of PM_2.5_ can effectively cover a range of PM_2.5_ concentrations at different confidence levels, providing more practical information for decision makers. Currently, a commonly adopted strategy for interval prediction is to use deep learning models for point prediction and then model the distribution of prediction errors [26]. Error distribution analysis usually takes the form of a probability density function. Parametric [27] and nonparametric methods [10] are the two main techniques for extracting the probability density function of the error distribution. Parametric methods require specific presuppositions about the error distribution, such as normal distribution, exponential distribution, etc. However, in practice, the error distribution may be skewed, and these assumptions may lead to bias in estimating the error distribution. In contrast, nonparametric methods are more flexible and adaptable as they do not require specific assumptions about the error distribution but rather infer the shape of the error distribution directly from the data. Among them, kernel density estimation (KDE) is a commonly used nonparametric method, which has been widely used in wind power generation interval prediction [28], wave height interval prediction [29], and other fields.

According to the above analysis of the literature, a long-term point-and-interval-prediction framework for PM_2.5_ concentration that integrates a convolutional neural network (CNN) and the Transformer model is introduced. The proposed approach comprehensively accounts for the interactions among air pollutants, meteorological factors, and PM_2.5_ data from strongly correlated stations. The main contributions of this study are as follows:

(1) In selecting influencing factors, this study considers both the interactions among various air pollutants and meteorological factors, as well as the correlations and synergies between monitoring stations across different geographic areas. The PM_2.5_ concentrations at strongly correlated stations are used as one of the features to mine the potential relationship between them and the target station.
(2) For long-term point prediction, this study notes the advantage of Transformer in mining the long-term dependence of time series. The overall structural design incorporates both CNN and Transformer models to effectively capture the long-term dependencies among multidimensional variables, thereby accomplishing stable and reliable PM_2.5_ predictions.
(3) In terms of long-term interval prediction, this study further utilizes KDE to obtain prediction intervals for PM_2.5_ concentration at different confidence levels based on point-prediction results to provide more information about uncertainty levels.

The rest of this paper is organized as follows. Section 2 provides an overview of related work. Section 3 reviews the theoretical principles of these methods. Section 4 presents the dataset and experimental results. Section 5 summarizes the discussion. Finally, Section 6 gives conclusions and outlines future work.

## 2. Related Works

### 2.1. Point Prediction

#### 2.1.1. Traditional Models

Traditional prediction models for PM_2.5_ can be roughly categorized into three types: deterministic methods [30], statistical methods [31], and machine learning methods [32]. Deterministic methods are a type of modeling based on the transport, dispersion, and chemical transformation processes of pollutants in the atmosphere. The most commonly used deterministic methods include the Community Multiscale Air Quality (CMAQ) model [30], the Weather Research and Forecasting (WRF) model [33], and others. However, these methods usually have limitations such as high computational complexity, parameter uncertainty, and high data requirements when confronted with complex atmospheric environments and PM_2.5_ concentration variations [18]. To address these challenges, statistical models have been proposed, such as the autoregressive integrated moving average (ARIMA) model [31], geographically weighted regression (GWR) [34], and the ridge regression (RR) model [35]. Unlike deterministic methods, statistical methods do not rely on complex theoretical understanding and provide a faster, simpler, and less costly implementation [36]. However, the prediction performance of statistical methods can be limited because they may struggle to effectively capture the complex nonlinear relationships between the data [9]. Therefore, for PM_2.5_ concentration prediction, some studies have chosen machine learning methods that can better handle the complex correlations between data. Common methods include the K-nearest neighbor algorithm (KNN) [37], random forest (RF) [18] and linear regression models [16]. These machine learning-based models capture the nonlinear relationships in the data more accurately. Nonetheless, they may struggle to capture long-term dependencies, leading to a rapid decrease in prediction accuracy as the time step of prediction increases.

#### 2.1.2. Deep Learning Models

In recent years, with the rapid development of artificial intelligence and big data technologies, deep learning has emerged as a pivotal area of research for air quality prediction [38,39,40]. Among them, the structure of the recurrent neural network (RNN) is well adapted to the highly nonlinear nature of air pollution data and is widely used in air quality prediction [41]. However, these RNN-based models face limitations, including gradient vanishing and time-consuming iteration propagation problems [42]. To overcome this limitation, LSTM, a variant of RNN, is gradually being applied to air pollution prediction. For instance, Zhang proposed a multi-scale PM_2.5_ prediction method based on bidirectional LSTM, yielding promising results in hourly PM_2.5_ prediction tasks in Beijing [43]. Gao et al. constructed a water quality parameter prediction model based on the results of driver analysis of an interpretable LSTM model [38]. The experimental results of these studies show that LSTM can alleviate problems such as RNN gradient drop. However, there are still limitations in capturing the long-term dependence of time series [44]. The Transformer model has recently been proposed to provide new ideas for time-series prediction [24]. For example, Li et al. applied the Transformer to time-series prediction and proposed an improvement scheme to solve the localization and memory bottleneck problems of the Transformer in time-series prediction applications, which laid a foundation for the subsequent advancements of the Transformer in this field [45]. Zuo et al. devised a Transformer-based THP model, leveraging self-attention mechanisms to capture long-term dependencies in event sequence data, thereby enhancing time-series prediction accuracy and computational efficiency [46]. Unlike traditional RNNs and LSTMs, the Transformer employs a self-attention mechanism that is independent of positional information, thereby enhancing its ability to capture information within lengthy sequences [45]. Currently, the Transformer and its variants remain among the advanced models for time-series prediction, especially in long sequence prediction applications. However, the above models, including Transformer, overlook the relationship between multiple variables. For instance, Zhang et al. have stated that the Transformer model makes it difficult to capture the correlations between different variable sequences in a multivariate time series [25]. However, time-series relationships between multivariate variables are crucial for time-series prediction. Many multivariate time-series prediction models have been developed to tackle this issue, with a majority adopting a hybrid modeling approach that combines two distinct modeling paradigms [47]. Hybrid approaches combine multiple prediction models, leveraging the strengths of each model structure, resulting in improved accuracy and stability. For example, Rick et al. constructed a deep learning architecture model combining LSTM and a CNN. LSTM was used to process time-series dependencies, and the CNN was used to capture spatial features in time-series data [28]. Experimental results demonstrated superior performance to traditional temporal convolutional network (TCN) methods in energy prediction tasks. Similarly, Kumar et al. introduced a multi-view CNN-BiLSTM model architecture for predicting time-series data of multiple pollutants in a highly polluted city. They demonstrated that it significantly outperformed traditional deep learning models in terms of performance [48]. CNN models can accurately extract local features [49] and can be used to extract relationships between long multivariable time series through sliding windows and convolution operations. Motivated by these insights, this study integrates the CNN and Transformer models for long-term prediction of PM_2.5_ concentration.

### 2.2. Interval Prediction

Compared with point prediction, interval prediction can provide a more accurate measure of the underlying uncertainty in the prediction [26]. Interval prediction results include upper and lower bounds that can be shown to be within a certain confidence level. Compared with point prediction, interval prediction provides more reliable and comprehensive information [10]. Currently, interval-prediction methods can be divided into two categories: directly predicting the upper and lower bounds of the intervals and estimating the probability density based on the point-prediction results [26]. Direct prediction methods often require the specification of a fixed interval width, making it challenging to calculate the uncertainty of prediction results [50]. Therefore, probabilistic prediction methods based on point-prediction results are more widely used for interval prediction [51]. These methods can be further categorized into non-parametric and parametric methods [27]. In practice, the difficulty in predetermining the accurate potential distribution of prediction errors makes the implementation of parametric methods challenging [29]. Non-parametric methods do not rely on specific error distribution assumptions and can accurately quantify the range of fluctuations [52], as in quantile regression (QR) [4] and KDE [28] methods. Xu et al. implemented forecasting of renewable energy generation and buildings’ electricity loads using quantile regression methods [53]. Li et al. proposed an hourly PM_2.5_ prediction system and utilized the KDE method to quantify the uncertainty of the prediction results [6]. Since the KDE method can directly provide the probability density function, it has become a more widely used probabilistic prediction method. For example, Niu et al. employed kernel density estimation with the Gaussian kernel function to obtain wind-power prediction intervals with different confidence levels and validated the method’s practicality and reliability through several experiments [28]. Among the various kernel functions of KDE, the Gaussian kernel function is mostly used for time-series prediction. The Gaussian kernel function exhibits a faster decaying tail, which aids in reducing the variance of the estimate and enhancing the accuracy of the density estimate. Therefore, the current study employs a Gaussian kernel function fitted to the KDE for long-term interval prediction of PM_2.5_ concentration.

## 3. Methodology

### 3.1. The Overall Framework

**Figure 1:**
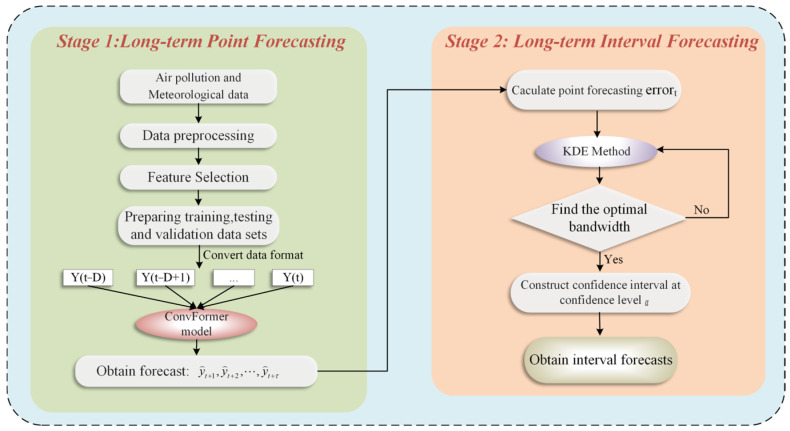
The overall framework of the proposed approach.

This study combines air pollutant and meteorological data from target stations and strongly correlated stations to exploit intricate spatial and temporal relationships for long-term point and interval prediction of PM_2.5_. The overall framework is depicted in Figure 1. First, multi-source data are collected and preprocessed. POI data in the study area are used to perform spatial clustering analysis of all monitoring stations to screen for strongly correlated stations. The Pearson correlation coefficient is used to analyze the correlation between all features to determine the feature variables for final input into the model. For model training and testing, the dataset is separated into three sets: training, validation, and test, in a 7:1:2 ratio. Second, a hybrid deep learning model based on a convolutional neural network and the Transformer is applied to achieve accurate long-term point predictions of PM_2.5_. Finally, KDE-based interval prediction is performed based on point-prediction error estimation to obtain the prediction intervals of PM_2.5_ at different confidence levels.

### 3.2. Preliminaries

Assume that there are N stations in the study area, denoted by the set $S=\{S_{1},S_{2},\cdots ,S_{N}\}$. Each station contains three attributes, including station id, longitude, and latitude. The count of different types of POIs around each station is denoted by $S^{*}\in R^{N\times K}$, where N denotes the number of stations and K denotes the total number of categories of POIs. Let $X_{i}\in R^{T*M}$ represent all the features of station $S_{i}$ at historical time T, encompassing air pollution data (PM_2.5_, PM_10_, CO, etc.), meteorological data (wind speed, wind direction, temperature, etc.), and PM_2.5_ concentration at strongly correlated stations. PM_2.5_ is the target pollutant in this study. For the target station $S_{i}$, the historical observation data $X^{\prime }\in R^{D*M}$ are used to predict the point and interval concentration of PM_2.5_ for the future time interval from t to t+τ, where D denotes the historical time step D∈{t−D,t−D+1,t}. The point prediction of PM_2.5_ concentration from t to t+τ is denoted by ${\hat{Y}}_{t+\tau }^{po\mathrm{int}}=\left\{{\hat{Y}}_{t+1}^{po\mathrm{int}},{\hat{Y}}_{t+2}^{po\mathrm{int}},\cdots ,{\hat{Y}}_{t+\tau }^{po\mathrm{int}}\right\},\;{\hat{Y}}_{t+\tau }^{po\mathrm{int}}\in R^{\tau *1}$. For a given confidence interval α, the interval prediction is denoted by ${\hat{Y}}_{\alpha ,t+\tau }^{\mathrm{int}erval}=\left[L_{\alpha ,t+\tau },U_{\alpha ,t+\tau }\right],\;{\hat{Y}}_{\alpha ,t+\tau }^{\mathrm{int}erval}\in R^{\tau *2}$.

### 3.3. Spatial Clustering Based on POIs

In order to examine the similarity and geographical association patterns across monitoring stations, this method obtains POI data for the study area using Baidu’s open API. The POI data include a range of geographical entities, such as business areas, cultural facilities, transportation hubs, and more. Afterwards, hierarchical clustering is utilized to spatially group all monitoring stations. Hierarchical clustering algorithms create a dendrogram by grouping into clusters stations that are both spatially close and similar in nature. This approach eliminates the requirement to pre-determine the number of clusters, making it easier to explore potential geographical patterns within the research region without prior information. Examining the clustering outcomes enhances our overall comprehension of the geographical connections between monitoring stations, uncovering groups of stations that display close spatial correlations with mutually beneficial changes. The spatial clustering module is represented by pseudo-code in Algorithm 1, and the formulas used in the algorithm are provided in Equations (1)–(3).
**Algorithm 1** Proposed spatial clustering approach     **Input:** $\;S=\{S_{1},S_{2},\cdots ,S_{n}\}\;(n\in 1,\cdots ,N);\;$$P=\{P_{1},P_{2},\cdots ,P_{m}\}\;(m\in 1,\cdots ,M).$$//S_{n},P_{m}$ represent station location information and POI information, respectively;     **Output:** C;  1:$S^{*}=\{S_{1}^{*},S_{2}^{*},\cdots ,S_{n}^{*}\}$$//\mathrm{initialize}\;S^{*}$ to a matrix of n×k$\;\mathrm{dimensions},\;S_{n}^{*}$ to a matrix of 1×k dimensions;  2:**for**$\;S_{i}$$\;\mathrm{in}\;\{S_{1},S_{2},\cdots ,S_{n}\}$ **do**  3:**for**$\;P_{j}$$\;\mathrm{in}\;\{P_{1},P_{2},\cdots ,P_{m}\}$ **do**  4:compute $\;d(S_{i},P_{j})$. according to Equation (1);  5:**if**$\;d(S_{i},P_{j})<1\;\mathrm{km}$ **do**  6:update $S_{i}^{*}$;  7:$C=\{C_{1},\cdots ,C_{n}\}$$//\mathrm{Each}\;S_{n}^{*}$ is regarded as a separate cluster;  8:**while** n>1 **do**  9:**for**$\;C_{i}$$\;\mathrm{in}\;\{C_{1},\cdots ,C_{n}\}$ **do**10:**for**$\;C_{j}$$\;\mathrm{in}\;\{C_{1},\cdots ,C_{n}\}$ **do**11:$M(i,j)=D(C_{i},C_{j})$ according to Equations (2) and (3);12:M(j,i)=M(i,j);13:find the most similar clusters: $C_{i^{*}}$$\;\mathrm{and}\;C_{j*}$;14:merge $C_{i^{*}}$$\;\mathrm{and}\;C_{j^{*}}$$:\;C_{i^{*}}=C_{i^{*}}\cup C_{j^{*}}$;15:**for** k=j* + 1, j* + 2, ⋯, n **do**16:$C_{k}=C_{k+1}$;17:n=n−1;18:return  C;

(1) $$d(S_{i},P_{j})=2\arcsin\sqrt{\sin^{2}\frac{\left(plat-slat\right)}{2}+\cos(plat)\times \cos(slat)\times \sin^{2}\frac{\left(p\ln g-s\ln g\right)}{2}}\times 6378.137$$

(2) $$dist(p_{a},q_{b})=\sqrt{{\sum_{k=1}^{n}\left(\frac{x_{k}^{\left(p_{a}\right)}-x_{k}^{\left(q_{b}\right)}}{s_{k}}\right)}^{2}}$$

(3) $$D(C_{i},C_{j})=\frac{1}{\left|C_{i}\right|\cdot \left|C_{j}\right|}\sum_{p_{a}\in C_{i},q_{b}\in C_{j}}dist(p_{a},q_{b})$$

where plng, plat denote the latitude and longitude of the POI and slng, slat denote the latitude and longitude of the monitoring stations. The value 6378.137 is the radius of the Earth’s equator in kilometers. $dist(p_{a},q_{b})$ represents the normalized Euclidean distance between data points $p_{a}$ and $q_{b}$, n represents the number of dimensions of the data point, $x_{k}^{\left(p_{a}\right)}$ and $x_{k}^{\left(q_{b}\right)}$, respectively, represent the values of data points $p_{a}$ and $q_{b}$ in the k dimension, $s_{k}$ is the standard deviation on the k dimension, $D(C_{i},C_{j})$ represents the similarity between clusters $C_{i}$ and $C_{j}$, and $\left|C_{i}\right|$ and $\left|C_{j}\right|$, respectively, represent the number of samples in each cluster.

### 3.4. ConvFormer Network

**Figure 2:**
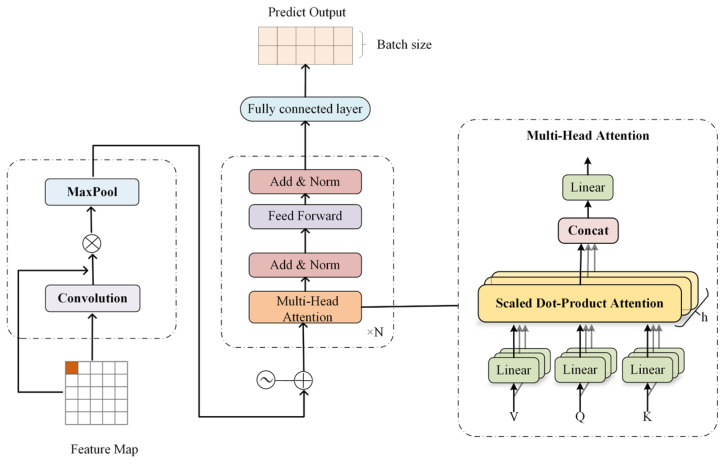
ConvFormer network architecture.

The structure of the proposed ConvFormer network is shown in Figure 2. The Transformer has a significant advantage in capturing long-term dependencies between time series. However, it is difficult for it to capture relationships between multivariate variables. Therefore, this study combines CNN to mine local patterns and short-term dependencies among multivariate variables and the Transformer to obtain long-term dependencies among time series. Additionally, this study adopts a direct multi-output strategy for long-term point prediction.

CNNs, representing a robust deep learning model, have demonstrated successful applications in image analysis, natural language processing, and various other fields. In the field of multivariate time-series prediction, a CNN automatically learns complex patterns and regularities in time-series data through its structure of convolutional and pooling layers, which can effectively deal with the interactions and temporal relationships among multiple variables. Therefore, this proposed method utilizes a CNN to process historical observation data. The input multivariate time-series data are converted into two-dimensional feature variables $X^{\prime }\in R^{D*M}$, and the convolution operation is performed to obtain the feature map $X^{\prime \prime }\in R^{D*M}$ where D denotes the sliding window step size and M denotes the dimension of the input features. The computation process of each element in the feature map is shown in Equation (4). Then, the maximum pooling operation is utilized to retain the most significant features in the multivariate data and ignore the less important information. The final matrix $X^{\prime \prime \prime }\in R^{D*1}$ is obtained as an input to the Transformer.

(4) $$X_{i,j}^{\prime \prime }=f_{conv}(\sum_{m=0}^{p}\sum_{n=0}^{q}w_{m,n}x_{i+m,j+n}^{\prime }+b)$$

where $X_{i,j}^{\prime \prime }$ denotes the feature output value of row i and column j of the feature map, $x_{i+m,j+n}^{\prime }$ denotes the value in row i+m and column j+n of the input feature matrix, $f_{conv}(\cdot )$ denotes the chosen activation function, $w_{m,n}$ denotes the weight value of the row m and column n of the convolution kernel, b denotes the deviation of the convolution kernel.

The Transformer model is a feed-forward neural network architecture. Its core is its self-attention mechanism, which can be utilized to effectively capture the relationship between any two points in a time series. In particular, the self-attention mechanism computes correlation weights between each position in the input sequence and other locations, and then applies these weights to generate a representation of each position. The self-attention mechanism is defined by the following formula:

(5) $$Attention(Q,K,V)=Soft\max(\frac{QK^{T}}{\sqrt{d_{k}}})V$$

where Q, K, V denote the query vector, key vector, and value vector, respectively, $d_{k}$ denotes the dimensionality of the key, and Softmax is the activation function that transforms the input to the interval [0, 1]. The self-attention mechanism derives the attention weights by evaluating the similarity between the query and the keys, and it produces the final representation through a weighted sum.

In contrast to the original Transformer architecture, this model eliminates the need for final probability calculations using Softmax. Instead, the final predicted value of the target pollutant concentration at the station at time t is derived by mapping the generated feature maps to the output values.

### 3.5. Interval-Prediction Method: Non-Parametric Kernel Density Estimation

Interval prediction of PM_2.5_ relies on point prediction, followed by the delineation of upper and lower bounds to define the prediction intervals. This approach quantifies the uncertainty in PM_2.5_ concentration changes, offering comprehensive early warning information for future PM_2.5_ variations. Non-parametric KDE is widely applied in interval prediction due to its independence from specific probability distribution assumptions. KDE, as a non-parametric estimation method, is not constrained by the specific form of probability distribution, which enables it to fit sample data accurately and reliably. Therefore, the proposed method uses KDE to quantitatively analyze and estimate point-prediction results for PM_2.5_. First, the error sequence $error=\left[error_{1},error_{2},\cdots ,error_{n}\right]$ is initially derived based on the difference between predicted and actual values within the training set. The optimal bandwidth $h_{opt}$ of the KDE is then determined based on a grid search and a five-fold cross-validation approach. Based on the obtained optimal bandwidth $h_{opt}$, the KDE model is fitted on the error sequence error, where the estimation function of KDE is described as follows:

(6) $$\overset{⌢}{f}(error)=\frac{1}{Nh_{opt}}\sum_{i=1}^{N}K\left(\frac{error-error_{i}}{h_{opt}}\right)$$

where N denotes the number of samples and K(⋅) denotes the kernel function. Commonly used kernel functions include the Gaussian kernel function, Epanechnikov kernel function, rectangular kernel function, etc. Compared with other kernel functions, the Gaussian kernel function can generate a smoother density estimation curve, which is conducive to capturing the overall characteristics of the data distribution. Therefore, the Gaussian kernel function is used here, and its expression is as follows:

(7) $$K\left(x\right)=\frac{1}{\sqrt{2\pi }}\exp\left(-x^{2}/2\right)$$

Based on the fitted KDE model, the probability density function (PDF) and cumulative distribution function (CDF) of the error are calculated. For a given confidence level α, the lower and upper bounds of the confidence interval are $l_{\alpha ,t+\tau }$ and $u_{\alpha ,t+\tau }$. Finally, the interval-prediction result ${\hat{Y}}_{\alpha ,t+\tau }^{\mathrm{int}erval}$ for the test set is obtained through Equation (8).

(8) $${\hat{Y}}_{\alpha ,t+\tau }^{\mathrm{int}erval}=\left[{\hat{Y}}_{t+\tau }^{po\mathrm{int}}+l_{\alpha ,t+\tau },{\hat{Y}}_{t+\tau }^{po\mathrm{int}}+u_{\alpha ,t+\tau }\right]$$

## 4. Experiment

### 4.1. Dataset Description and Preprocessing

#### 4.1.1. Description

**Figure 3:**
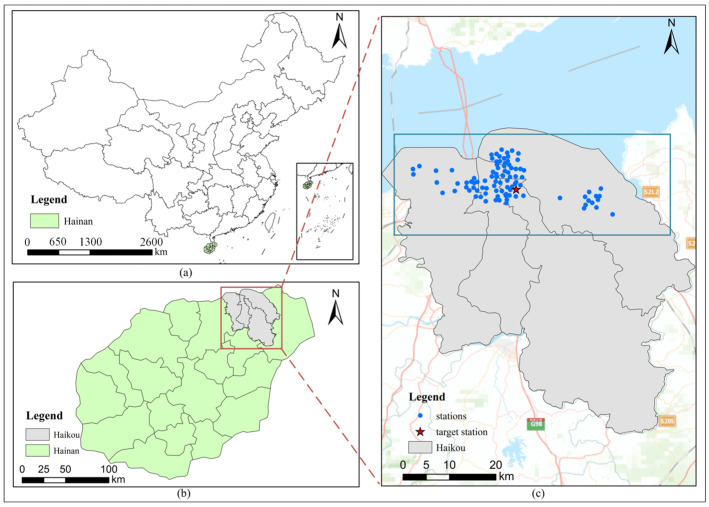
Study area and spatial distribution of air monitoring stations: (**a**) The green part is the boundary map of Hainan province, (**b**) The gray part is the boundary map of Haikou city, (**c**) Distribution of air monitoring stations in Haikou (zoom figure).

The study area selected was Haikou City, located in Hainan Province. Initially, air pollution concentration data (PM_2.5_, PM_10_, O_3_, CO, etc.) and meteorological data (wind speed, temperature, pressure, etc.) for the same period were collected from the monitoring stations in Haikou. The dataset on air quality included hourly data spanning from 30 October 2020 to 26 December 2023. The distribution of monitoring stations is marked in blue in Figure 3. The target station for this study was S9, which is indicated by the red marking in Figure 3. S9, being in the heart of the city and surrounded by a multitude of stores, provides a better response to the influence of spatial–temporal correlation on the model’s predictions and is relatively representative. Additionally, the POI data obtained through Baidu’s open API included 14 different categories, as shown in Table 1. Each POI point contained six attributes, from which first-level classification, longitude, and latitude were selected for the application, resulting in a total of 92,108 POIs.

#### 4.1.2. Dataset Preprocessing

**Figure 4:**
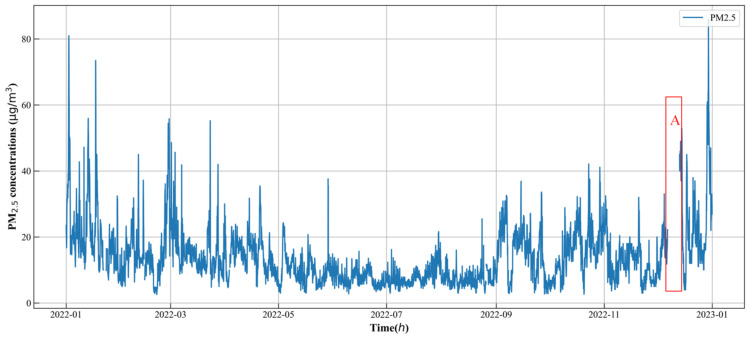
Time series of hourly PM_2.5_ at the S9 station and a large number of missing values in time period A.

Figure 4 depicts the hourly PM_2.5_ concentration sequence from the target station in 2022. The figure shows that the PM_2.5_ concentration exhibited significant volatility and instability. Notably, a continuous missing value was evident, represented by the red mark labeled A in the figure. Data gathering can be hindered by problems including equipment breakage and transmission faults, which can result in outliers and missing results. These missing values can negatively affect the prediction of PM_2.5_ concentration. Thus, preprocessing was necessary before constructing the prediction model. The forward filling method was specifically employed to address missing values within short time periods (e.g., up to 4 h) in the original data. For medium and long time periods (e.g., more than 4 h but less than 72 h), the multiple interpolation method was utilized to fill in the missing values. However, missing values in long time periods (e.g., exceeding 72 h) were simply deleted and handled accordingly. In order to eliminate the effect of magnitude between different features, a normalization method was finally used to scale the data within the values (0, 1).

After data preprocessing, a total of 26,451 data samples were obtained. The dataset was separated into the training set, validation set, and test set according to the ratio 7:1:2. The training set contained 18,526 sample points for model training and parameter optimization. The validation set consisted of 2635 sample points to assess the training and generalization performance of the model. The test set included 5290 sample points to validate and evaluate the long-term prediction performance of the model. The statistical descriptions of the training, validation, and test sets are shown in Table 2.

### 4.2. Evaluation Metrics

#### 4.2.1. Evaluation Metrics of Point Prediction

To comprehensively evaluate the performance of the proposed approach in this study, three evaluation metrics including root mean square error (RMSE), mean absolute error (MAE), and R-square (R^2^) were used. These metrics were calculated as shown in Equations (9)–(11). RMSE denotes the sample standard deviation of the difference between predicted and observed values. MAE denotes the mean of absolute errors between predicted and observed values. The MAE is a linear score in which all individual differences are equally weighted on the mean. RMSE penalizes high variance more compared with MAE. R^2^ was used to assess the fitting ability of the model, with values closer to 1 indicating a better fit of the predicted learning results.

(9) $$RMSE=\sqrt{\frac{1}{N}\sum_{t=1}^{N}{\left(y_{t}-{\overset{⌢}{y}}_{t}\right)}^{2}}$$

(10) $$MAE=\frac{1}{N}\sum_{t=1}^{N}\left|y_{t}-{\overset{⌢}{y}}_{t}\right|$$

(11) $$R^{2}=1-\frac{\sum_{t=0}^{N}{\left(y_{t}-{\overset{⌢}{y}}_{t}\right)}^{2}}{\sum_{t=0}^{N}{\left(y_{t}-\bar{y}\right)}^{2}}$$

where N denotes the total number of samples, $y_{t}$ denotes the true value, ${\overset{⌢}{y}}_{t}$ denotes the predicted value, and $\bar{y}$ denotes the mean value.

#### 4.2.2. Evaluation Metrics of Interval Prediction

A good interval-prediction model should ensure that observations align with the prediction interval to the closest degree possible. At the same time, these prediction intervals should be as narrow as possible to improve the accuracy of the prediction. Therefore, in order to comprehensively assess the performance of the interval-prediction model proposed in this study, prediction interval coverage probability (PICP) and prediction interval normalized averaged width (PINAW) metrics were used. These metrics were calculated as shown in Equations (12)–(14). PICP is used to reflect the probability that an actual observation falls within the prediction interval at a given confidence level. It serves as a key metric for assessing the efficacy of an interval-prediction model. PINAW is employed to reflect the normalized average width of all prediction intervals. Typically, smaller PINAW values indicate narrower prediction intervals, i.e., higher accuracy.

(12) $$PICP=\frac{1}{N}\sum_{i=1}^{N}C_{i}^{\alpha }$$

(13) $$C_{i}^{\alpha }=\left\{\begin{array}{l} 0,\;Y_{i}^{po\mathrm{int}}\notin \left[L_{\alpha ,i},U_{\alpha ,i}\right]\\ 1,\;Y_{i}^{po\mathrm{int}}\in \left[L_{\alpha ,i},U_{\alpha ,i}\right] \end{array}\right\}$$

(14) $$PINAW=\frac{1}{N\left(\max(Y^{po\mathrm{int}})-\min(Y^{po\mathrm{int}})\right)}\sum_{i=1}^{N}\left(U_{\alpha ,i}-L_{\alpha ,i}\right)$$

where N represents the number of samples, α represents the confidence level, $Y^{po\mathrm{int}}$ and ${\overset{⌢}{Y}}_{i}^{po\mathrm{int}}$ represent the actual observed and predicted values of the point prediction, respectively, $C_{i}^{\alpha }$ represents a Boolean value, where 1 indicates that the observation falls within the prediction interval, and 0 otherwise, $U_{\alpha ,i}$ and $L_{\alpha ,i}$ represent the upper and lower bounds of the prediction interval at the confidence level α, respectively.

### 4.3. Feature Selection

**Figure 5:**
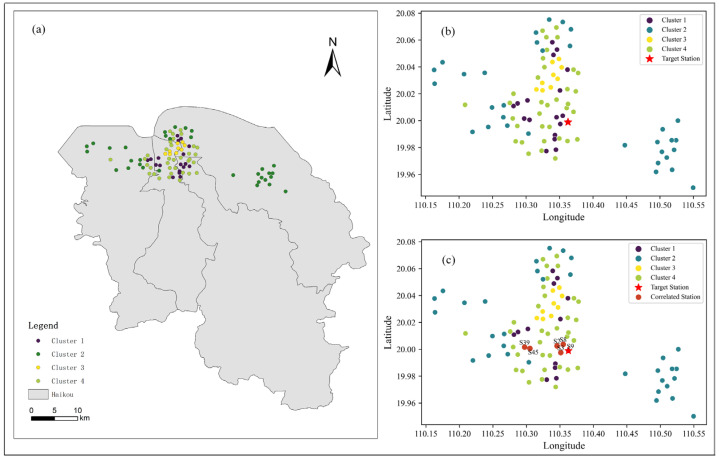
The selection results of the correlated stations: (**a**,**b**) Spatial clustering results based on POIs, (**c**) Results of spatial anisotropy analysis: stations S2, S3, S8, S39, and S45 are strongly correlated with the target station.

The hierarchical clustering method and POI data were employed to cluster all 95 monitoring stations, resulting in four distinct clusters, as illustrated in Figure 5. The stations in Cluster 1 and Cluster 3 were located in the city center, characterized by dense architectural facilities, such as prominent urban structures such as commercial buildings, cultural institutions, and retail centers. The stations in Cluster 2 were located in the outside region, predominantly flanked by educational institutions and recreational areas. The stations in Cluster 3 were located in central locations, with a convergence of various activity facilities nearby. According to the clustering results, it was observed that the target monitoring station (S9) was part of Cluster 1. The stations in Cluster 1 underwent additional refinement to identify those that correlated with the target station, in conjunction with spatial variability analysis. Figure 5c illustrates the selection of stations that were highly correlated, such as S2, S3, S8, S39, and S45. Ultimately, the model’s prediction was informed by the PM_2.5_ concentrations from these stations that were highly correlated.

**Figure 6:**
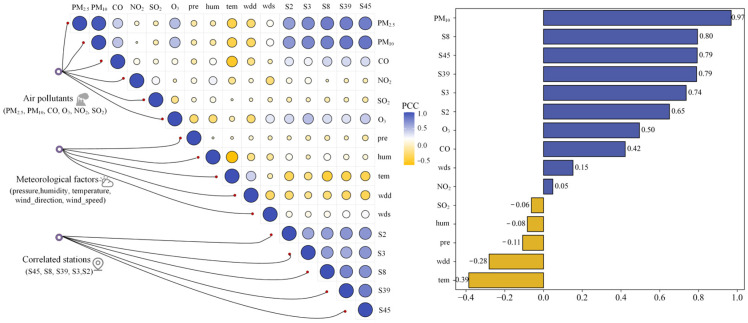
Correlations between the influencing factors.

Considering that redundant features negatively affect the model performance, Pearson’s correlation coefficient was further used in this study to analyze the influences of air pollutant concentration and meteorological factors on PM_2.5_ concentration at strongly correlated stations. The results are presented in Figure 6. Pearson’s correlation coefficient ranges from −1 to 1, where 1 indicates a perfect positive correlation, −1 indicates a perfect negative correlation, and 0 indicates no linear correlation between the variables. The final results of the calculations are displayed in Figure 1, revealing the strongest correlation between PM_2.5_ and PM_10_ at the target station, followed by the selected strongly correlated stations. Notably, NO_2_, SO_2_, and humidity exhibited weak correlations with PM_2.5_, with correlation coefficients $\left|r\right|<0.1$. Therefore, this study excluded NO_2_, SO_2_, and humidity from the prediction modelling process.

### 4.4. Point-Prediction Performance Analysis

To verify the effectiveness of ConvFormer for long-term prediction of PM_2.5_, this study analyzed the predictive performance of six baseline models (ns_Transformer [54], Informer [55], Autoformer [56], Reformer [57], Pyraformer [58], and LightTS [59]) in comparison with the proposed approach, validating its effectiveness. These baseline models currently achieve good results in long-term series prediction tasks. Comparison with these models enabled a better assessment of the performance advantages of the ConvFormer model in the task of long-term prediction of PM_2.5_.

The chosen station for the experiment was S9. Experiments were performed to predict the PM_2.5_ concentration at the station for time intervals of 24, 48, and 96 h. The accuracy of the prediction models was assessed by employing metrics such as MAE, RMSE, and R^2^. These metrics measured the disparity between the observed and predicted values. The experimental results are summarized in Table 3. In summary, the ConvFormer model had superior performance in predicting PM_2.5_ levels, with the lowest errors in terms of RMSE and MAE, and the highest R^2^ score. Specifically, compared with the baseline models, in the prediction tasks of t+24, t+48, and t+96, the MAE of ConvFormer decreased by 8.44%, 9.31%, and 8.13% on average, the RMSE was reduced by 7.94%, 10.89%, and 9.12% on average, and the R^2^ increased by 10.29%, 26.2%, and 27.47% on average. Among the baseline models, the ns_Transformer, Informer, Autoformer, and Reformer models are modifications based on the Transformer, and perform well in long-term prediction tasks [24]. However, in our air pollution prediction experiments, the Transformer model significantly outperformed these models.

**Figure 7:**
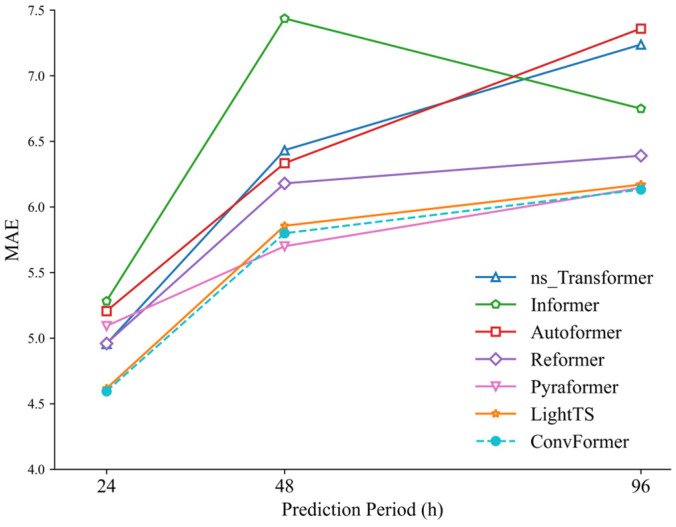
MAE of ConvFormer and the baseline models.

**Figure 8:**
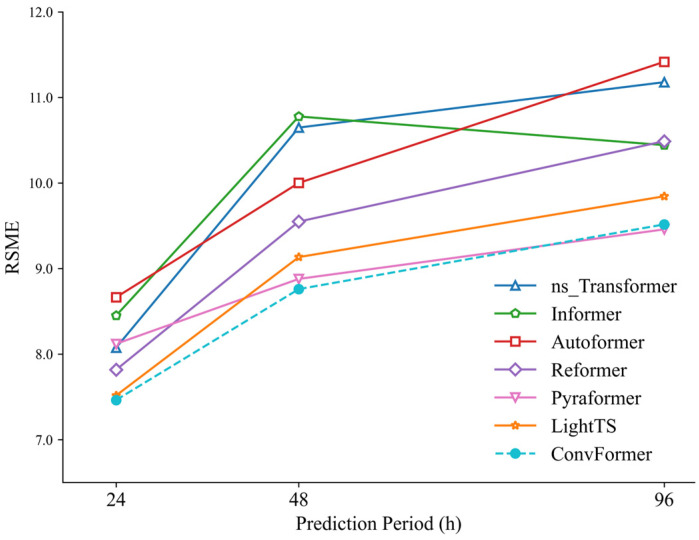
RMSE of ConvFormer and the baseline models.

**Figure 9:**
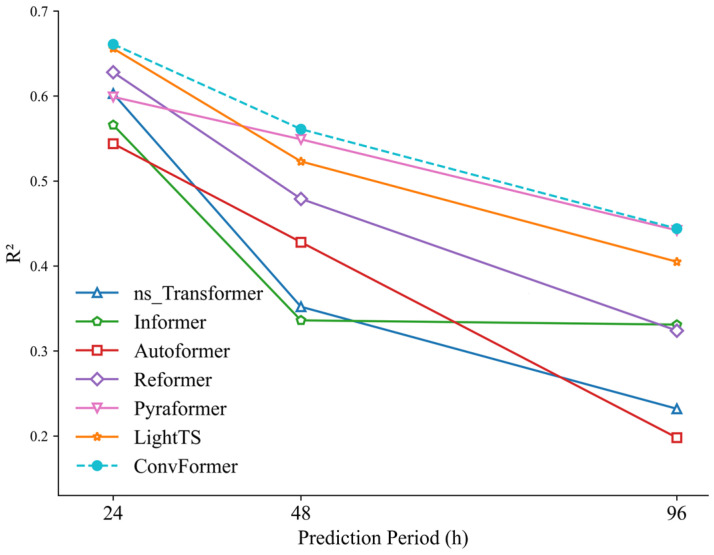
R^2^ of ConvFormer and the baseline models.

Figure 7, Figure 8 and Figure 9 visually illustrate the changes in MAE, RMSE, and R^2^ values for each model in prediction tasks over different time periods. It is evident from these figures that the prediction accuracies of all models decreased as the prediction time increased. One possible explanation for this phenomenon is that as the prediction time lengthened, it became increasingly challenging for the models to capture the long-term dependencies between historical time-series information, thus affecting prediction accuracy.

### 4.5. Interval-Prediction Performance Analysis

**Figure 10:**
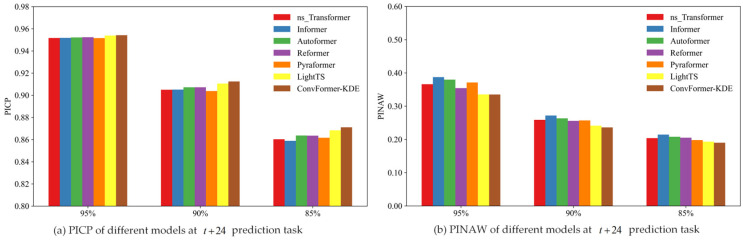
The interval-prediction performance of different models at t+24 prediction task.

**Figure 11:**
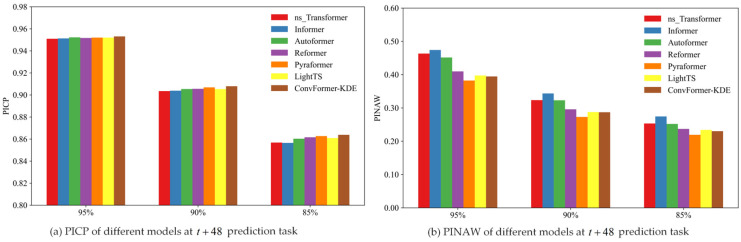
The interval-prediction performance of different models at t+48 prediction task.

**Figure 12:**
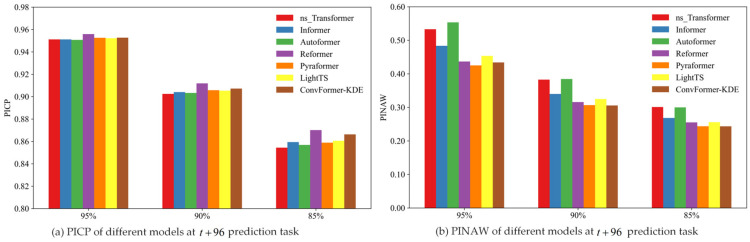
The interval-prediction performance of different models at t+96 prediction task.

In order to compare the interval-prediction effectiveness of different models based on the KDE method, this study compared the interval-prediction results of different models using the same training set and test set. The experimental results are summarized in Table 4. For the various point-prediction models, the PICP of all models exceeded the preset confidence level at different confidence levels and prediction times. Figure 10, Figure 11 and Figure 12 depict the PICP and PINAW values for each model under different prediction t tasks. Specifically, the ConvFormer-KDE model achieved the highest PICP and the lowest PINAW in the t+24 prediction task at the same confidence level. PICP measures the proportion of actual observations in the prediction interval, typically ranging from 0 to 1, with values closer to 1 indicating more accurate predictions that cover more actual observations. PINAW measures the width of the prediction intervals. Combining PICP and PINAW can assess the overall performance of interval prediction. As a result, in the t+24 prediction task, the ConvFormer-KDE demonstrated the best performance, making it able to provide more accurate PM_2.5_ information for the public and air pollution prevention workers. In the t+48 and t+96 prediction tasks, the ConvFormer-KDE also achieved relatively high PICP and PINAW. It is worth noting that while the Reformer ranked in the middle of all models in terms of predictive performance in the t+96 point-prediction task, it achieved the best PICP values at all confidence levels in the t+96 interval-prediction task. However, it did not have the lowest PINAW. This suggests that the Reformer model was overly conservative in the t+96 prediction task, resulting in extensive prediction intervals. Although this ensured a high PICP, the prediction uncertainty remained high. Therefore, overall, the ConvFormer-KDE demonstrated the best interval-prediction performance.

### 4.6. The Result of Ablation Experiment

To further evaluate the effectiveness of ConvFormer-KDE in this study, the employed network modules (e.g., convolutional neural networks and transformers) were thoroughly tested to assess their effectiveness in extracting input features. In the experiments, the proposed method was compared with a single CNN and Transformer model, respectively. During the experiment, the input variables of the three models remained consistent. The experimental results are shown in Table 5 and Table 6. The results showed that the combination of CNN and Transformer improved the prediction learning performance compared with relying only on the CNN or Transformer for prediction learning. Specifically, in terms of point prediction, the ConvFormer-KDE improved the R^2^ by 2.16%, 4.66%, and 8.78%, respectively, compared with CNN in the prediction tasks of t+24, t+48, and t+96. Compared to Transformer, the ConvFormer-KDE improved the R^2^ by 4.09%, 15.67%, and 26.85% for the prediction tasks of t+24, t+48, and t+96. In terms of interval prediction, the performance of ConvFormer-KDE was generally superior that of the CNN and Transformer models at different confidence levels. Therefore, the experimental results demonstrate the effectiveness of the ConvFormer-KDE model combining a CNN with the Transformer, indicating that it is highly capable of predicting the long-term PM_2.5_ concentration with significant advantageous performance.

## 5. Discussion

This study proposes a prediction framework based on the ConvFormer-KDE model, which combines CNN, Transformer, and KDE techniques to obtain long-term point and interval prediction for PM_2.5_ concentration. In selecting influencing factors, some meteorological factors and other pollution factors cannot be ignored. Therefore, PM_10_, CO, O_3_, wind speed, temperature, pressure, and wind direction, which are highly correlated to PM_2.5_, were included in the modelling of this study. In addition, PM_2.5_ values from stations that were strongly correlated with the target station were used as input to the model. In the modelling process, unlike previous studies where the final prediction results are obtained after integrating the prediction results of two separate models, this study converts the CNN-extracted features into the input dimensions required by the Transformer model. Subsequently, the Transformer is utilized to mine the long-term dependencies in the time series to obtain the prediction results. The ConvFormer-KDE takes full advantage of different deep-learning modules. CNN is able to learn temporal relationships and interactions between multivariate variables, and the multi-attention mechanism of the Transformer enables the model to track each data point in relation to another specific data point, allowing it to capture long-term dependencies between temporal sequences. In terms of output strategy selection, this method employs direct output of multiple predicted duration values simultaneously rather than recursively training multiple models. The key to recursive multistep prediction methods lies in continuously updating the dataset and utilizing the updated dataset to make predictions. These methods have the problem of error accumulation becoming worse as the prediction time increases, since each prediction builds on the previous one. The direct multi-output approach chosen in this study can alleviate this problem, and the model structure is simpler and more efficient in terms of computational efficiency.

In terms of interval prediction, directly predicting the upper and lower bounds of intervals often necessitates specifying a fixed interval width, making it challenging to calculate the uncertainty of prediction results. Therefore, implementing interval prediction based on point-prediction results is more widely used. The point-prediction model used in this study plays an important role in interval prediction. A preliminary analysis of the point-prediction error is performed and a probability density function (PDF) of the point-prediction error is constructed using the KDE method. Subsequently, the cumulative distribution function (CDF) is employed to depict the distribution of the error at a specific confidence level, and the upper and lower bounds of the interval prediction are ultimately derived at the designated confidence level. In KDE, the selection of the kernel function holds significant importance as it directly impacts the level of smoothing and the bias–variance trade-off of the estimation. In this study, the Gaussian function was selected as the kernel function for KDE. Typically, the Gaussian function offers smoother characteristics compared with other kernel functions, resulting in a more continuous and smoother distribution of weights within the observations and yielding a smaller bias.

## 6. Conclusions and Future Directions

### 6.1. Summary of Experimental Results

This study proposes a method that combines a CNN, the Transformer model, and kernel density estimation to achieve long-term point and interval prediction of PM_2.5_ concentration. The effectiveness and stability of this model were verified with data from Haikou. Compared with a range of baseline models, including ns_Transformer, Informer, Autoformer, Reformer, Pyraformer, and LightTS, the experimental results showed that the ConvFormer-KDE provided results that were closer to the actual values and the prediction performance was better than the baseline models. The experimental results and analysis demonstrated that the ConvFormer-KDE performed better on the task of long-term point and interval prediction of PM_2.5_, with good prediction generalization ability and robustness, providing a new direction for PM_2.5_ prediction.

### 6.2. Caveats and Future Directions

There are still some limitations in this study. The ConvFormer-KDE in this study requires a large amount of data for training to adequately capture long-term dependencies in sequences. Therefore, the model’s prediction performance with small quantities of sample data is poor. Secondly, this study applied the proposed approach only to PM_2.5_ concentration prediction, and the ability to make accurate long-term predictions of multiple air pollutants simultaneously is an important target for future research. Future research will focus on the above research directions, and the model proposed in this study will play an important role in these follow-up works.

## Figures and Tables

**Table 1:** POI categories.

First Level Classification	Second Level Classification
Education and Training	Higher education institutions, secondary schools, elementary schools, kindergartens, adult education, parent–child education, special education schools, study abroad agencies, research institutions, training institutions, libraries, science and technology centers, others.
Medical	General hospitals, specialist hospitals, clinics, pharmacies, medical centers, sanatoriums, emergency centers, disease control centers, others.
Transportation Facilities	Parking lots, service areas, bus stations, wharves, train stations, ferries, toll stations, airports, coach stations, others.
Sports and Fitness	Stadiums, extreme sports venues, fitness centers, others.
Tourist Attractions	Town squares, zoos, botanical gardens, amusement parks, museums, aquariums, heritage sites, churches, scenic spots, others.
Finance	Banks, ATMs, credit unions, investment banking, pawnshops, others.
Automobile Services	Automobile sales, automobile repair, automobile cosmetics, automobile parts, automobile rental, automobile inspection yard, others.
Life Services	Logistics, public toilet, post office, salons, hairdressers, bath and massage, laundry, public utilities, others.
Food	Chinese restaurants, foreign restaurants, snack and fast-food restaurants, cake and dessert stores, cafes, cafeterias, bars, and others.
Hotels	Star hotels, fast hotels, apartment hotels, others.
Shopping	Shopping centers, department stores, supermarkets, convenience stores, home building materials, home appliances and digital stores, bazaars, duty-free stores, others.
Leisure and Entertainment	Resorts, open farms, cinemas, karaoke halls, theaters, dance halls, Internet cafes, gaming arcades, bath and massage, leisure plazas, others.
Company Enterprise	Companies, factories, others.
Real Estate	Office buildings, residential areas, dormitories, neighborhoods, villages, community centers, others.

**Table 2:** Statistical descriptions of different data sets.

Data Set	Numbers	Maximum	Minimum	Mean	Standard Deviation
Train set	18,526	103.48	2.53	15.84	11.52
Validation set	2635	78.20	4.0	19.45	9.66
Test set	5290	97.0	2.62	16.83	12.78

**Table 3:** Comparison of the performance of different point-prediction models.

Model	t+24			t+48			t+96		
	MAE	RMSE	R^2^	MAE	RMSE	R^2^	MAE	RMSE	R^2^
ns_Transformer	4.956	8.075	0.603	6.432	10.648	0.352	7.237	11.179	0.232
Informer	5.282	8.45	0.566	7.436	10.778	0.336	6.749	10.443	0.331
Autoformer	5.206	8.664	0.544	6.333	10.001	0.428	7.359	11.417	0.198
Reformer	4.960	7.817	0.628	6.179	9.549	0.479	6.390	10.489	0.324
Pyraformer	5.092	8.120	0.599	**5.700**	8.879	0.549	6.146	**9.458**	0.442
LightTS	4.616	7.516	0.656	5.856	9.134	0.523	6.170	9.845	0.405
ConvFormer	**4.595**	**7.463**	**0.661**	**5.799**	**8.760**	**0.561**	**6.132**	**9.516**	**0.444**

**Table 4:** Comparison of the performance of different interval-prediction models under different confidence levels.

Confidence Levels	Model	t+24		t+48		t+96	
		PICP	PINAW	PICP	PINAW	PICP	PINAW
α=95%	ns_Transformer	0.9517	0.3661	0.9510	0.4633	0.9512	0.5328
	Informer	0.9518	0.3874	0.9513	0.4740	0.9512	0.4835
	Autoformer	0.9522	0.3798	0.9523	0.4515	0.9507	0.5535
	Reformer	0.9524	0.3544	0.9517	0.4097	**0.9559**	0.4368
	Pyraformer	0.9516	0.3713	0.9520	**0.3823**	0.9526	**0.4251**
	LightTS	0.9539	0.3352	0.9520	0.3974	0.9522	0.4535
	ConvFormer-KDE	**0.9542**	**0.3351**	**0.9531**	**0.3946**	**0.9527**	**0.4340**
α=90%	ns_Transformer	0.905	0.2589	0.9035	0.3234	0.9025	0.3827
	Informer	0.9051	0.2718	0.9039	0.3435	0.9041	0.3400
	Autoformer	0.9072	0.2634	0.9054	0.3230	0.9033	0.3846
	Reformer	0.9072	0.2558	0.9056	0.2958	**0.9119**	0.3159
	Pyraformer	0.9038	0.2572	0.9069	**0.2730**	0.9058	0.3067
	LightTS	0.9106	0.2414	0.9053	0.2876	0.9053	0.3248
	ConvFormer-KDE	**0.9125**	**0.2361**	**0.9079**	**0.2871**	**0.9072**	**0.3057**
α=85%	ns_Transformer	0.8603	0.2039	0.8568	0.2532	0.8544	0.3011
	Informer	0.8589	0.2146	0.8565	0.2745	0.8594	0.2685
	Autoformer	0.8637	0.2080	0.8603	0.2519	0.8569	0.3000
	Reformer	0.8636	0.2051	0.8616	0.2371	**0.8701**	0.2552
	Pyraformer	0.8617	0.1980	0.8627	**0.2192**	0.8589	0.2437
	LightTS	0.8684	0.1933	0.8609	0.2340	0.8606	0.2558
	ConvFormer-KDE	**0.8711**	**0.1900**	**0.8638**	**0.2300**	**0.8663**	**0.2434**

**Table 5:** The results of ablation experiments for point prediction.

Model	t+24			t+48			t+96		
	MAE	RMSE	R^2^	MAE	RMSE	R^2^	MAE	RMSE	R^2^
CNN	4.671	7.623	0.647	**5.706**	9.014	0.536	6.247	9.851	0.405
Transformer	4.79	7.745	0.635	5.871	9.492	0.485	6.603	10.282	0.350
ConvFormer-KDE	**4.595**	**7.463**	**0.661**	**5.799**	**8.760**	**0.561**	**6.132**	**9.516**	**0.444**

**Table 6:** The results of ablation experiments for interval prediction.

Confidence Levels	Model	t+24		t+48		t+96	
		PICP	PINAW	PICP	PINAW	PICP	PINAW
α=95%	CNN	0.9532	0.3336	0.9521	0.3922	0.9518	0.4561
	Transformer	0.9525	0.3335	0.9531	0.4027	0.9514	0.4641
	ConvFormer-KDE	**0.9542**	**0.3351**	**0.9531**	**0.3946**	**0.9527**	**0.4340**
α=90%	CNN	0.9088	0.2399	0.9048	0.2848	0.9042	0.3292
	Transformer	0.9077	0.2388	0.9080	0.2813	0.9037	0.3256
	ConvFormer-KDE	**0.9125**	**0.2361**	**0.9079**	**0.2871**	**0.9072**	**0.3057**
α=85%	CNN	0.8650	0.1907	0.8582	0.2355	0.8591	0.2577
	Transformer	0.8636	0.1918	0.8643	**0.2252**	0.8570	0.2576
	ConvFormer-KDE	**0.8711**	**0.1900**	**0.8638**	**0.2300**	**0.8663**	**0.2434**

## Data Availability

The datasets generated and analyzed during the current study are not publicly available but are available from the corresponding author on reasonable request.
